# CT Perfusion as a Selection Tool for Mechanical Thrombectomy, a Single-Centre Study

**DOI:** 10.5334/jbsr.1944

**Published:** 2020-01-20

**Authors:** Emilie De Muynck, Vincent Huybrechts, Dimitri Hemelsoet, Elisabeth Dhondt, Peter Vanlangenhove, Luc Defreyne

**Affiliations:** 1 Master Students Medical Sciences, UGent University, BE; 2 Department of Neurology, Ghent University Hospital, BE; 3 Department of Vascular and Interventional Radiology, Ghent University Hospital, BE

**Keywords:** ischemic CVA, CT perfusion, mechanical thrombectomy, anterior circulation

## Abstract

**Background:**

Recently, CT perfusion (CTP) has been proposed as a selection tool for stroke patients to be treated with endovascular thrombectomy. We investigated whether functional outcome following endovascular treatment was improved after the introduction of CTP.

**Methods:**

This retrospective single-centre study includes all patients with a major vessel occlusion in the anterior circulation that received a CTP and underwent a mechanical thrombectomy from 2014 up to 2015. CTP were visually evaluated. Demographics, stroke and time data, procedural data, functional outcomes as measured by the modified Rankin Scale (mRS) and the association between these variables were studied. A comparison was made with the results of a similar local retrospective study from before the CTP “era”.

**Results:**

Eighty-nine patients were included in this study. Median National Institutes of Health Stroke Scale (NIHSS) was 16 (Interquartile range 6). At three months, good functional outcome (GFO; mRS 0–2) was achieved in 48.4% and excellent functional outcome (EFO; mRS 0–1) in 34.4% of patients. The mortality rate at three months was 14.5%. GFO at one year was 44.8%, EFO was 31.3% and mortality 21.1%. The duration of the thrombectomy procedure and the EFO were associated (p = 0.032). The outcome improvement achieved with CTP was higher compared to the reference study (GFO 48.4% versus 44%; EFO 34.4% versus 29%) but remained below the statistical significance.

**Conclusions:**

Mechanical thrombectomy for anterior circulation strokes based on CTP did not result in a significant functional outcome improvement. The duration of the thrombectomy procedure was the sole time-interval related to improved functional outcome.

## Introduction

Five randomized clinical trials demonstrated a beneficial effect of endovascular therapy in anterior circulation stroke patients [1,3,4,5]. The standard technique to select patients for a mechanical thrombectomy consists of a CT angiography, proving a major vessel occlusion. CT perfusion (CTP) is another technique that can be used for this selection. CTP provides physiological information of the infarcted area and the surrounding penumbra that remains potentially viable. CTP may differentiate between patients in whom recanalization would be futile and patients with a significant penumbra to be rescued. CTP has found general acceptance but the extent to which it can improve patient selection is still debated. We wanted to explore whether patients’ outcomes had changed after the introduction of CTP. The presented study was conducted retrospectively in a group of stroke patients receiving thrombectomy after CTP following the study period used in an early report of thrombectomy outcome [6].

## Methods and Materials

### Study design

This single-centre study was designed to examine whether the outcome of mechanical thrombectomy would improve when based on CTP. Between January 1st 2014 and the 31st of December 2015, all patients with a major vessel occlusion in the anterior circulation treated by mechanical thrombectomy after CTP were included. This study was conducted retrospectively, comprising a time period following a period used in an early multicentric study from the same region without CTP selection. The present study was approved by the local ethics committee (B670201630032 & B670201630033).

### Inclusion criteria

All patients who suffered from acute ischemic stroke in the anterior (internal carotid artery [ICA] and middle cerebral artery [MCA]) circulation who had a thrombectomy were included. CT angiography was required to prove a major vessel occlusion. Treatment with IV rtPA before starting an endovascular intervention was allowed.

### Exclusion criteria

There was no age limit, nor were there specified exclusion criteria for comorbidity. No patients were excluded based on the severity of the stroke, as measured with the National Institutes of Health Stroke Scale (NIHSS). A six-hour time window for non-wake-up strokes was applied, although a deviation was allowed on a case-by-case basis. For wake-up strokes, onset time was not available and therefore no window could be applied.

Patients who did not receive a CTP scan were excluded from this study. The interventional radiologist and neurologist decided on the basis of this CTP if the patient was eligible for a mechanical thrombectomy. If there was a mismatch on the CTP with at least approximately 1/3 of the affected area supposed to be penumbra, then the patient received thrombectomy.

### Procedure of the mechanical thrombectomy

A Solitaire® stent retriever (Medtronic) was used for the mechanical thrombectomy. Patients who were treated before May 2015 were put on heparin anticoagulation during the intervention. From May 2015 on, patients no longer received heparin. Patients were administered local anaesthesia, except when unstable or restless. In those cases, we decided to perform the thrombectomy under general anaesthesia with intubation.

### Data collection

The following patient data were obtained from medical records: age; sex; location of the occlusion; treatment with rtPa; NIHSS (pre-procedural); mRS at 3 and 12 months; time between symptom onset and procedure start, the moment of incision (“onset-to-groin”); time between symptom onset and reperfusion (“onset-to-reperfusion”); the duration of the endovascular procedure and the use of a pre-procedural MRI (perfusion and diffusion) in patients with a WUS. Patients with a WUS were not included in analysis of the “onset-to-groin” and “onset-to-reperfusion” intervals. The time of procedure start was retrieved from the anaesthesiologist’s report.

### Neuro-imaging analysis

The modified treatment in cerebral infarction (mTICI score) was used to measure the reperfusion grade post thrombectomy (Table 1).

**Table 1:** mTICI (modified treatment in cerebral infarction) score.

Grade	Angiographic definition

0	no reperfusion
1	antegrade reperfusion past the initial occlusion, but limited distal branch filling with little or slow distal reperfusion
2a	antegrade reperfusion of less than half of the occluded target artery previously ischemic territory (e.g. in one major division of the middle cerebral artery (MCA) and its territory)
2b	antegrade reperfusion of more than half of the previously occluded target artery ischemic territory (e.g. in two major divisions of the MCA and their territories)
3	complete antegrade reperfusion of the previously occluded target artery ischemic territory, with absence of visualised occlusion in all distal branches

### Comparison with the non-CTP study

We compared the presented results with those of anterior circulation thrombectomy as described in the study of Fockaert et al. [1]. The non-CTP study took place in the same region as our study; but in a time before CTP was implemented as a selection method in the acute stroke protocol.

### Outcome

The modified Rankin Scale (mRS) was used to measure functional outcome. The primary endpoint of this study was a good functional outcome (GFO) defined as mRS 0–2 at 90 days, obtained by the treating neurologist not blinded for the result of the mechanical thrombectomy. Secondary outcomes were clinical significant haemorrhage, excellent functional outcome (EFO) defined as mRS 0–1 at 90 days, mortality at 90 days and GFO, EFO and mortality at one year. Intracranial haemorrhage was determined on the CT scan 24 hours after the endovascular procedure.

### Statistical analysis

The location of the occlusion was dichotomized into the anterior circulation: ICA and/or MCA. The mTICI scores were dichotomized into a substantial reperfusion (mTICI 2b–3) versus low reperfusion (mTICI 0–2a). P-values ≤ 0.05 were considered significant. Univariate and multivariate analysis (with GFO and EFO as outcome variables) were performed. Wake-up strokes were included in the multivariate analysis; but not the intervals “onset to groin” and “onset to reperfusion” as parameter. SPSS24.0 was used to perform statistical analysis [2].

## Results

Eight-nine patients with an anterior ischemic stroke underwent a mechanical thrombectomy. Two patients did not consent and were excluded. Eleven patients did not receive a CTP or the CTP could not be interpreted due to patient movement or contrast leakage. These patients were excluded.

We report on 76 patients who had an occlusion in the anterior circulation: 3 ICA (3.9%), 65 MCA (85.5%) and 8 (10.5%) with a tandem (ICA + MCA) occlusion. Baseline characteristics are summarized in Table 2.

**Table 2:** Baseline characteristics of all included patients.

Parameter		Overall (N = 76)	ICA (N = 3)	MCA (N = 65)	Tandem (N = 8)

Age (years)	Median (IQR)	69.5 (19.5)	63 (–)	70 (22)	72.5 (18)
Gender – no. (%)	Man	31 (40.8)	1 (33.3)	25 (38.5)	5 (62.5)
	Woman	45 (59.2)	2 (66.7)	40 (61.5)	3 (37.5)
NIHSS score	Median (IQR)	16 (6)	17 (–)	16 (6)	19(4)
Stroke cause – no (%)	Large artery atherosclerosis	3 (3.9)	0 (0)	2 (3,1)	1 (12.5)
	Cardio-embolism	41 (53.9)	1 (33.3)	34 (52.3)	6 (75)
	Other determined etiology	7 (9.2)	0 (0)	7 (10.8)	0 (0)
	Undetermined etiology	25 (32.9)	2 (66.7)	22 (33.8)	1 (12.5)
Intravenous rtPA – no (%)		59 (77.6)	2 (66.7)	50 (76.9)	7 (87.5)
Time onset to groin (min)	Median	260	285	260	220
Time onset to reperfusion (min)	Median	356	379	355	371
Duration thrombectomy (min)	Median	90	94	89	86
Wake-up stroke (%)		12 (15.8)	0 (0)	10 (15.4)	2 (25)
Intravenous heparin		19 (25)	0 (0)	17 (26.2)	2(25)

ICA internal carotid artery, MCA medial middle cerebral artery, NIHSS National Institutes of Health Stroke Scale.

**Figure 1:**
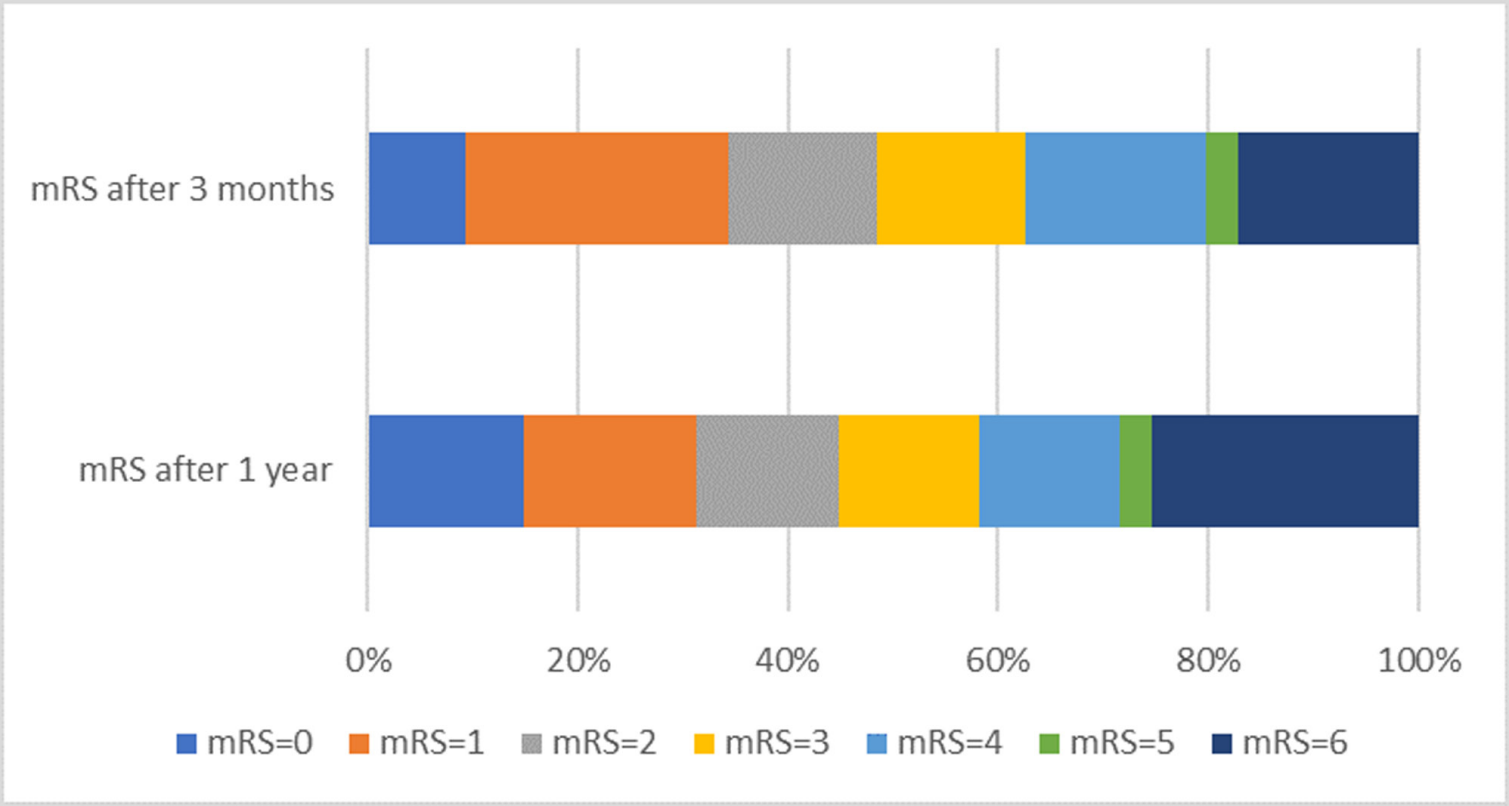
Modified Rankin Scale Scores at 90 days and at 1 year.

Data of the functional outcome at three months was obtained from 64 (84.2%) patients. Thirty-one of the 64 patients (48.4%) had a GFO and 22 (34.4%) had an EFO. The mortality rate at three months was 14.5% (11 of 76 patients). Data regarding intracranial haemorrhage was obtained in 74 patients (97.4%). Ten patients had an intracranial haemorrhage (13.5%); three of those were of clinical significance (4.1% of all patients) (Figure 1).

In 63 of the 64 patients (98.4%) without a WUS, the time interval from onset-to-groin was documented. Median onset-to-groin interval was 260 minutes. 8 (12.5%) patients had an interval greater than six hours. The time interval from onset-to-reperfusion was documented in 63 of 64 cases (98.4%). Median onset-to-reperfusion interval was 356 minutes. The duration of the procedure was known in all 76 cases; the median time was 90 minutes (range 37/289).

No significant association could be found between the onset-to-groin interval and the GFO, EFO or mortality at three months. Respective p-values were 0.569; 0.800 and 0.940. After eliminating patients with a time window of six hours or more, the non-association between the onset-to-groin interval and the GFO, EFO or mortality at three months remained (Respective p-values were 0.559; 0.992 and 0.677).

A significant association was found between the onset-to-reperfusion interval and GFO (p = 0.042). If the duration was shorter, then more patients had a GFO. No significant association was found with mortality (p = 0.553) and EFO (p = 0.107).

A significant association was found between the procedure duration and the EFO (p = 0.032). There were more patients with an EFO if the duration of the procedure was shorter (p = 0.074). No significant association was found regarding GFO or mortality (p = 0.063).

GFO and EFO did not differ between WUS and non-WUS (p = 0.268; p = 0.490). A similar number of intracranial haemorrhages were found in WUS (16.7%) and non-WUS (12.9%) (p = 0.661). Five of the twelve people with a WUS received an MRI pre-procedure.

**Figure 2:**
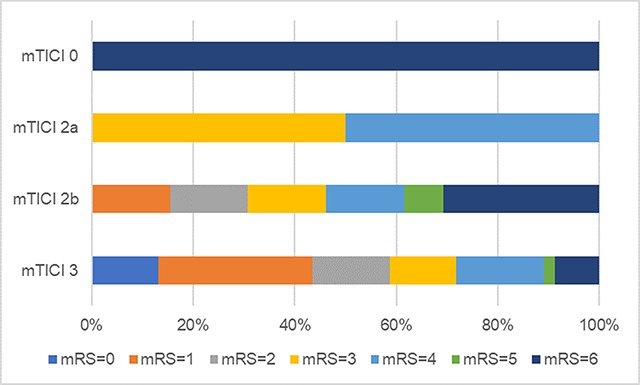
Modified Rankin Scale at three months categorized by mTICI score.

A substantial reperfusion (mTICI 2b or 3) was achieved in 68 of 76 (89.5%) patients. Five patients had a mTICI0, 3 mTICI2a; 17 mTICI2b and 51 mTICI3. No significant association was found between the outcome and the achievement of a substantial reperfusion. P-values were: 0.053 for GFO; 0.155 for EFO and 0.085 for mortality. There were no patients who did not have a substantial reperfusion but did have a GFO or EFO (Figure 2).

There was no significant association between the administration of IV thrombolysis and the occurrence of a clinical significant haemorrhage (p = 1.000). All patients with a clinical significant haemorrhage had received IV thrombolysis.

In a multivariate model on GFO, we added the variables age, sex, administration of rtPA, NIHSS before treatment, the time of the procedure, reperfusion, general anaesthesia, heparin administration and parenchymal hematoma. The variables that significantly predicted GFO were administration of rtPA (p = 0.019), time of the procedure (p = 0.006), administration of heparin (p = 0.009) and NIHSS before treatment (p = 0.004). In the multivariate analysis for EFO the only variable that could be retained in the final model was NIHSS before treatment (p = 0.009). A lower NIHSS before treatment was a predictor of an EFO.

Functional outcome at one year was obtained from 67 (88.2%) patients. Thirty (44.8%) had a GFO and 21 (31.3%) had an EFO. The mortality at one year was 22.4% (17 of 76 patients). There was no significant difference between the number of patients with a GFO at three months and at one year (p = 1.000). No difference was found between the number of patients with the EFO at three months and at one year (p = 1.000).

### Comparison with non-CTP study

The majority of demographics, stroke characteristics and outcome in the present study and the study of Fockaert et al. are comparable (Table 3).

**Table 3:** Comparison of demographics and results of our study and the part of study of Fockaert et al. studying occlusions in the anterior circulation.

	Present study (n = 76)	Fockaert et al. (n = 65)

Demographics		
Median age	69.5	64
Substantial reperfusion	89.6%	72%
Sex (male)	40.8%	34%
Median NIHSS on admission in hospital	16	15
rtPA	77.6	60
Onset to groin	260	252
Outcome		
GFU	48.4%	44%
EFU	34.4%	29%
Mortality	14.5%	22%
Significant ICH	3 (4,1%)	4 (5%)

## Discussion

In patients with a stroke by a major vessel occlusion and who had undergone CTP and thrombectomy, GFO at three months was 48.4% and EFO was 34.4%. In this selected group of stroke patients, the mortality at three months was 14.5%, and 4.1% of patients had a significant ICH.

CTP was introduced to select patients with a higher probability to benefit from a mechanical thrombectomy. Additionally, by evaluating CTP, we would also be able to exclude patients from a thrombectomy procedure when major infarction was established and penumbra limited. However, the outcome improvement compared to a similar retrospective multicentric study that did not use CTP, was only mild (GFO 48.4% versus 44%; EFO 34.4% versus 29%) [1]. This monocentric study was conducted in the same area, so that the presented study can be considered as a sequel of the original but with additional CTP as a selection method.

The explanation why the expected better outcome at three months was not observed is multifactorial. Firstly, the time period of inclusion reflects the early days of experience with CTP. CTPs were visually read out by the radiologist and communicated to the neurologist and interventional radiologist. The interpretation was done in consensus, but the infarct volume and penumbra were not quantified. The penumbra threshold was visually determined by 1/3 of the affected area (on CBF and CBV). Therefore, evaluation of CTP was not yet standardized and probably interpreted in a way to offer maximum chances to a maximum of patients, leading to an overoptimistic inclusion.

Secondly, the work-flow around the CTP and its interpretation could have increased the onset-to-groin time. This seems not the case, as the onset-to-groin time was similar in the retrospective study without CTP (260 vs. 252 minutes) [1].

In a meta-analysis performed by Ryu et al. mechanical thrombectomy resulted in a higher number of GFO in patients selected by CTP (51.1% versus 45.6%; p = 0.06) [8]. However, significancy was only observed for a subgroup of patients with a specified onset time (OR 1.3; p < 0.01), and not for patients with an unknown onset time (OR 1.08; p = 0.66). From this review, it could not be concluded that CTP was of benefit for all candidates for thrombectomy. It is remarkable that for unknown time of onset (or wake up) strokes the use of CTP did not result in a selection of patients that would benefit most. In the presented study, strokes with known and unknown onset were included. The presented GFO results lie between the GFO of the “intervention” group with CTP and the control group of the meta-analysis [8].

Five RCTs showed that mechanical thrombectomy in stroke patients for an occlusion in the anterior circulation leads to a better functional result than the standard treatment [3,4,5,6,7]. The study that used a CTP for the selection of patients was the study with the highest percentage of GFO (EXTEND-IA: 71%). The CTP was processed with the use of fully automated software (RAPID) [6]. A quantitative analysis of the infarct core and penumbra seems more efficient for selection of good candidates for thrombectomy. An automated analysis of the images with standardised algorithms (either Philips, Siemens, Olea or RAPID) is now implemented in our department, in combination with the clinical NIHSS score.

Mortality in the presented study was lower than in the non-CTP study (14.5% versus 22%) [1]. Whether CTP was adequate to exclude patient with a malignant MCA infarction, hence could have reduced mortality, is not clear. We have not registered the patients with an unfavourable CTP. Also, a higher reperfusion rate (TICI3 and 2B of 89.6% compared with 72%) and a higher percentage of patients receiving rtPA (77.6% compared with 60%) in the presented study could have reduced infaust outcomes 1). A recent study regarding the role of intravenous thrombolysis (IVT) prior to mechanical thrombectomy, did not find IVT to be an independent predictor of good functional outcome, low mortality or high recanalization rates [9].

In our study, no association was found between onset-to-groin time and the mRS outcome at three months. The time to reperfusion, however was significantly associated with GFO at three months but not with EFO at three months. Subsequently, one could calculate and expect that the duration of the procedure would be associated with the mRS outcome. This was only true for the EFO at three months, and not for the GFO. A distinct time between the onset of symptoms and procedure start has always been used as a selection criterium for IV and endovascular therapy [10]. Why this was not found in the presented study might be explained by a variation in the registered time period. For this study, procedure start was retrieved from the anaesthesiologist’s report, which might not always be identical to the puncture time. Also possible is that by introducing CTP in the selection algorithm, the value of “time” as a predictor of functional outcome has diminished. In studies that did not use CTP, a shorter “onset-to-groin” has been found to be strongly associated with a better functional outcome at three months [9]. When CTP was used as a selection method, this association was not found [10]. The shorter the thrombectomy procedure the better is the expected outcome. Longer procedure duration can result in complications due to anaesthesia or manipulations [11]. Patients with a longer procedural duration are less likely to have a complete recanalization [12]. In our department we decided after the study that the mechanical thrombus retrieval should be done within two hours, otherwise the procedure will be stopped.

There was no significant difference in outcome to be found between WUS and non-WUS. This can signify that CTP might be a good selection method for decision making on endovascular stroke treatment in patients with an unknown time of symptom onset. A recent study by Caruso confirmed that CTP is a good method for selecting WUS patients who could benefit from endovascular therapy [13].

A substantial reperfusion (TICI III and 2B) was achieved in 89.6% of patients. This excellent result was comparable with reperfusion rates observed in RCTs. In contrast to these RCTs, a significant increase in patients with GFO at three months was not found. Other RCTs did report higher percentage of good mRS scores at three months: e.g. SWIFT PRIME (88% reperfusion; 60% GFO) and EXTEND-IA (86% reperfusion; 71% GFO) [5,7]. It might be that the substantial reperfusion rate in the presented study is overestimated while only based on the radiological reports. In other studies [1,3,4,5,6,7], mTICI scores were read out blinded in by two independent raters. Also very few patients in our study had a low reperfusion rate; thus limiting the statistical power. It should be noted that not a single person with “low reperfusion” had a GFO at three months.

There are some limitations to this study. It is conducted retrospectively, with a relative low number of patients. The drop out ratio because of unknown mRS score at three months (15.8%) or because of a WU stroke (15.7%) is substantial. There was no quantitative analysis of CTP (infarct core and penumbra) and no blinded analyses of the post thrombectomy reperfusion (TICI).

Our study has some clear strengths. Every patient received a CTP prior to endovascular treatment and no hard exclusion criteria regarding comorbidities or age were applied. Thus, the results represent a real life experience for endovascular treatment. The study took place in a major centre for invasive stroke treatment, in a region of another early report of intracranial thrombectomy, so comparison of outcome evolution after introduction of CTP is possible.

## Conclusion

CTP with visual analysis as a decision-making method only marginally improved selection of good candidates for a mechanical thrombectomy in anterior circulation strokes. To determine the added value of a CTP, it should probably be examined in a quantitative way and in a RCT. Based on the result of our study that shorter procedure duration was associated with a better outcome, we recommend limiting all future endovascular thrombus retrievals to two hours.

## Figures and Tables

**Table:** 

Abbreviation	Explanation

CTP	CT perfusion
RCT	randomized controlled trial
GFO	good functional outcome
EFO	excellent functional outcome
NIHSS	National Institutes of Health Stroke Scale
mTICI score	modified treatment in cerebral infarction
mRS	modified Rankin Scale
ICA	internal carotid artery
MCA	middle cerebral artery
rtPA	recombinant tissue plasminogen activator
WUS	wake-up stroke
CBF	cerebral blood flow
CBV	cerebral blood volume

## References

[1] Fockaert N, Coninckx M, Heye S, Defreyne L, Brisbois D, Goffette P. Mechanical endovascular thrombectomy for acute ischemic stroke: A retrospective multicenter study in Belgium. Acta Neurologica Belgica. 2016; 116: 7–14. DOI: 10.1007/s13760-015-0552-7 26445955

[2] IBM Corp. Released 2016 IBM SPSS Statistics for Windows, Version 24.0 Armonk, NY: IBM Corp.

[3] Jovin TG, Chamorro A, Cobo E, de Miquel MA, Molina CA, Rovira A. Thrombectomy within 8 hours after symptom onset in ischemic stroke. N Engl J Med. 2015; 372: 2296–3. DOI: 10.1056/NEJMoa1503780 25882510

[4] Berkhemer OA, Fransen PS, Beumer D, et al. A randomized trial of intraarterial treatment for acute ischemic stroke. N Engl J Med. 2015; 372: 11–20. DOI: 10.1056/NEJMoa1411587 25517348

[5] Campbell BC, Mitchell PJ, Kleinig TJ, et al. Endovascular therapy for ischemic stroke with perfusion-imaging selection. N Engl J Med. 2015; 372: 1009–18. DOI: 10.1056/NEJMoa1414792 25671797

[6] Goyal M, Demchuk AM, Menon BK, et al. Randomized assessment of rapid endovascular treatment of ischemic stroke. N Engl J Med. 2015; 372: 1019–30. DOI: 10.1056/NEJMoa1414905 25671798

[7] Saver JL, Goyal M, Bonafe A, et al. Stent-retriever thrombectomy after intravenous t-PA vs. t-PA alone in stroke. N Engl J Med. 2015; 372: 2285–95. DOI: 10.1056/NEJMoa1415061 25882376

[8] Ryu WHA, Avery MB, Dharampal N, Allen IE, Hetts SW. Utility of perfusion imaging in acute stroke treatment: A systematic review and meta-analysis. J Neurointerv Surg. 2017; 9: 10 DOI: 10.1136/neurintsurg-2016-012751 PMC5890922 28899932

[9] Weber R, Nordmeyer H, Hadisurya J, et al. Comparison of outcome and interventional complication rate in patients with acute stroke treated with mechanical thrombectomy with and without bridging thrombolysis. J Neurointerv Surg. 2017; 9: 229–233. DOI: 10.1136/neurintsurg-2015-012236 26902926

[10] Saver JL, Goyal M, van der Lugt A, et al. Time to treatment with endovascular thrombectomy and outcomes from ischemic stroke: A meta-analysis. JAMA. 2016; 316: 1279–10. DOI: 10.1001/jama.2016.13647 27673305

[11] Turk AS, Nyberg EM, Chaudry MI, et al. Utilization of CT perfusion patient selection for mechanical thrombectomy irrespective of time: A comparison of functional outcomes and complications. J Neurointerv Surg. 2013; 5: 518–22. DOI: 10.1136/neurintsurg-2012-010452 22935349

[12] Spiotta AM, Vargas J, Turner R, Chaudry MI, Battenhouse H, Turk AS. The golden hour of stroke intervention: Effect of thrombectomy procedural time in acute ischemic stroke on outcome. J Neurointerv Surg. 2014; 6: 511–6. DOI: 10.1136/neurintsurg-2013-010726 24014466

[13] Caruso P, Naccarato M, Furlanis G, et al. Wake-up stroke and CT perfusion: Effectiveness and safety of reperfusion therapy. Neurol Sci. 2018; 39(10): 1705–1712. DOI: 10.1007/s10072-018-3486-z 29987433

